# Gender-based violence during COVID-19 among adolescent girls and young women in Nairobi, Kenya: a mixed-methods prospective study over 18 months

**DOI:** 10.1136/bmjgh-2021-007807

**Published:** 2022-02-24

**Authors:** Michele R Decker, Kristin Bevilacqua, Shannon N Wood, Grace Wamue Ngare, Mary Thiongo, Meagan E Byrne, Anaise Williams, Bianca Devoto, Nancy Glass, Lori Heise, Peter Gichangi

**Affiliations:** 1 Department of Population, Family and Reproductive Health, Johns Hopkins University Bloomberg School of Public Health, Baltimore, Maryland, USA; 2 Bill and Melinda Gates Institute for Population and Reproductive Health, Johns Hopkins University, Baltimore, Maryland, USA; 3 Department of Sociology, Gender and Development Studies, Kenyatta University, Nairobi, Kenya; 4 Women’s Economic Empowerment Hub, Kenyatta University, Nairobi, Kenya; 5 International Centre for Reproductive Health Kenya, Nairobi, Kenya; 6 Johns Hopkins University School of Nursing, Baltimore, Maryland, USA; 7 Department of Public Health and Primary Care, Ghent University Faculty of Medicine and Health Sciences, Ghent, Belgium

**Keywords:** COVID-19, public health, child health

## Abstract

**Introduction:**

Adolescent girls and young women (AGYW) disproportionately experience gender-based violence (GBV), which can increase during emergencies like the COVID-19 pandemic.

**Methods:**

A cohort of youth ages 15–24 in Nairobi, Kenya was surveyed at three time points over an 18-month period prior to and during the COVID-19 pandemic: June–August 2019 (prepandemic), August–October 2020 (12-month follow-up) and May 2021 (18-month follow-up). We characterise (1) prevalence, relative timing and help-seeking for leading forms of GBV, (2) GBV trajectories over 18 months and (3) associations of individual, dyad and COVID-related factors on GBV trajectories among AGYW (n=612) in Nairobi, Kenya. Virtual focus group discussions (n=12) and interviews (n=40) contextualise quantitative results.

**Results:**

Intimate partner violence (IPV) prevalence hovered at 17% across time points (ever at pre-pandemic; past 12 months at 12-month follow-up (2020); past 6 months at 18-month follow-up (2021)); non-partner sexual violence (SV) was 3% at 12-month and 18-month follow-up. Overall, 27.6% of AGYW experienced IPV during the pandemic. IPV during the pandemic was associated with work as the primary pre-COVID activity, low social support and partner age difference >4 years. Among AGYW partnered at all three time points, 66.2% stayed IPV-free (no IPV), 9.2% saw IPV resolve by 18-month follow-up, while 11.1% had IPV start and 13.6% experienced intermittent IPV. Help-seeking for IPV and SV in 2020 (11.1% and 4.6%, respectively) increased to 21.7% and 15.1%, respectively, by 2021. Qualitative results speak to impacts of curfews, and pandemic-related financial stress in prompting conflict and threatening traditional gender roles, and underlying conditions that enable IPV.

**Conclusion:**

The persistence of IPV against AGYW in Nairobi prior to and during the COVID-19 pandemic reflects endemic conditions and pandemic-specific stressors. Youth, including unmarried youth, remain a priority population for GBV prevention and survivor-centred response.

**Key questions**

**What is already known?**

- Global evidence suggests increases in gender-based violence (GBV) among adult women following COVID-19 mitigation efforts implemented beginning in early 2020.
- Women in Nairobi, Kenya are highly affected by GBV; increases in household tension and conflict among adult women have been reported since the start of the COVID-19 pandemic, as have modest increases in household violence and increases in violence outside the home.
- Adolescent girls and young women, including unmarried youth, are disproportionately affected by GBV, yet remain poorly understood with regard to COVID-19-related impacts; longitudinal data on risk trajectories are lacking.

**Key questions**

**What are the new findings?**

- In this cohort of urban adolescent girls and young women (AGYW), prevalence of intimate partner violence (IPV) was consistent at approximately 17% across three time points prior to and during the COVID-19 pandemic (ever at prepandemic (2019); past 12 months at 12-month follow-up (2020); past 6 months at 18-month month follow-up (2021)); non-partner sexual violence was less pervasive (3%) at 2020 and 2021 time points.
- IPV trajectories illustrate movement both in and out of relative relationship safety.
- Heightened risk for IPV during COVID-19 was identified among AGYW engaged in the work force pre-COVID, those with low social support, and those in relationship with older partners (>4 years).
- Qualitative data contextualise results and articulate the impact of curfews and financial stressors, as well as underlying conditions that enable abuse independent of the pandemic.

**What do the new findings imply?**

- This first study to characterise COVID-relative IPV trajectories among AGYW in Nairobi confirms persistent risk both prior to and during the COVID-19 pandemic.
- Both primary prevention and survivor-centred support services are needed to meet the unique needs of AGYW and overcome the stigma of accessing care.
- The disruption of the pandemic may afford a window to reset harmful underlying social norms and gendered social systems that perpetuate violence against young women with impunity.

## Background

Gender-based violence (GBV) threatens health and human rights, with most severe consequences including injury and death.^1 2^ Globally, one in three women experience physical and/or sexual violence (SV) by a partner or non-partner in their lifetime,^3^ and intimate partner violence (IPV) is responsible for over one-third of women’s homicides.^4^ Accordingly, eliminating GBV features among the United Nations Sustainable Development Goals, alongside solutions to achieve gender equality.^5^ Crisis and its aftermath increase risk for GBV, while undermining women’s economic and social standing.^6–8^ The COVID-19 pandemic immediately raised concerns for escalated GBV due to economic disruption and subsequent household/relationship stressors, together with social and travel restrictions.^8–10^ Increased GBV since COVID-19 has been documented in many settings,^11^ likely reflecting limited mobility, social isolation, increased time in the home with potential abusers, and financial and social stress that enable conflict. Simultaneously, mobility restrictions, lack of privacy and fears of transmission can create new barriers to violence-related help-seeking.^9^

Adolescent girls and young women (AGYW) are an under-recognised yet critical population for GBV prevention and response. Interpersonal violence is the fifth-leading source of disability-adjusted life-years lost among youth ages 10–24 globally.^12^ Adolescence represents the age at onset of IPV,^13^ and experiencing IPV early in life increases risk for adulthood victimisation.^14 15^ The impact of IPV and non-partner SV on subsequent reproductive health, educational attainment and financial earnings^16 17^ make it important to characterise and mitigate violence in this population. As seen in the Ebola outbreak, AGYW are not spared the GBV-related impact of epidemics and other public health crises.^18^ Longitudinal research on violence trajectories is extremely limited among AGYW, and it remains unclear how pandemic-related mobility restrictions and other stressors influence risk trajectories among youth, particularly those who are not cohabitating. Trajectory data are essential to understand persistence of GBV over time at the individual level, identify risks associated with new GBV, and estimate how many (and how) individuals experiencing GBV transition into safety; together these data can refine interventions.

In Kenya, as in many settings, GBV was prevalent prior to the COVID-19 pandemic, with an estimated 27.2% of AGYW ages 15–24 reporting past 12-month physical or sexual IPV.^19^ National data illustrate that intimate partners are primary perpetrators of SV among young women,^20^ cautioning against a false dichotomy of IPV and SV. The pandemic appears to have exacerbated GBV in Kenya; women report feeling less safe at home since the start of the pandemic.^21^ The national domestic violence hotline reported a 1000% increase in calls between February and June, 2020^22^ and reports of SV have similarly increased at outpatient visits.^23^ The Council on the Administration of Justice described a ‘significant spike’ in SV offences at the start of the pandemic.^24^ Such reports spurred a government probe in 2020 to investigate rising GBV.^22^

In their 2021 Research Round Up,^11^ the Center for Global Development identified 74 studies published on GBV related to the COVID-19 pandemic to date. Among those, two studies were conducted in Kenya among adult populations and four with young adults in other settings, with mixed results. In Nairobi, increases in household tension and conflict were reported, as were modest increases in household violence and increases in violence outside the home; perpetrator(s) were not specified.^25^ In rural Kenya, risk of domestic violence was variable but statistically stable over 11 weeks following the onset of COVID-19 mitigation restrictions.^26^ Among the four studies that focused on youth in settings outside of Kenya, authors found high levels of familial conflict,^27 28^ and increased potential for online violence and harassment.^29^ More recent research illustrates declines in relationship quality due to COVID-19 restrictions, which elevate IPV risk.^30^ To our knowledge, ours is the first study to focus on experiences of GBV among AGYW in Kenya during COVID-19 and examine violence trajectories beginning with a pre-COVID-19 baseline.

Against this backdrop, this study examines (1) prevalence and help-seeking related to leading forms of GBV, (2) trajectories from prepandemic to 18-month follow-up and (3) associations of individual, dyad and COVID-related factors on GBV trajectories among a cohort of AGYW in Nairobi, Kenya, first recruited in 2019. Results provide timely evidence to guide safety planning and supports for youth during the remainder of the pandemic, recovery investments that respond to the safety needs of AGYW, and insight into the needs of this population in future emergencies.

## Methods

### Study population

This study is embedded in the Nairobi Youth Respondent Driven Sampling Survey (YRDSS), an ongoing cohort study of adolescents and young adults, and draws on data from three time points over an 18-month period prior to and during the COVID-19 pandemic. The YRDSS began recruitment in June–August 2019 using respondent-driven sampling (RDS), a chain-based recruitment method that begins with purposefully selected seeds, followed by monitored peer-to-peer coupon distribution.^31^ RDS is designed to recruit harder-to-reach populations, such as urban youth.^31^ Eligible youth were age 15–24 years, unmarried and residing in Nairobi for at least 1 year. Further details are elsewhere.^32^ The 2019 pre-pandemic survey round recruited 1357 participants; of whom 95% (1293/1357) consented for recontact and provided contact information. This cohort was recontacted and surveyed in August–October 2020 (12-month follow-up; n=1,217 (94% retention)), and in April–May 2021 (18-month follow-up; n=1,177 (97% retention)) for the purpose of characterising gender-related impacts of the pandemic, including, but not limited to, GBV. Our team designed and implemented this study; data are publicly available.^33–35^

### Data collection

Trained resident enumerators (REs) conducted data collection in either English or Swahili using OpenDataKit on tablets or smart phones. Data collection at pre-pandemic baseline occurred via self-administered survey at community-based sites; due to the COVID-19 restrictions, 2020 and 2021 rounds were conducted by phone with added privacy protections. REs are trained in sensitive data collection concerning family planning and sexual activity, and received specialised training specific to GBV protections. They are ages 28–41 years to minimise intergenerational conversations on sensitive topics. All data were collected in accordance with best practices for GBV research,^36^ including specialised interviewer training, privacy protections and provision of support resources. To ensure privacy and safety for GBV and other sensitive topics during remote data collection, the audio privacy protocol inquired about safety and audio privacy before beginning data collection with the option to reschedule, and provided a ‘safe phrase’ for the respondent to discreetly signal a privacy breach during data collection. The survey length was kept minimal to limit the total time participants spent to avoid arousing suspicion or concern about their use of phone or their whereabouts. Participants were instructed that they could skip any question they did not wish to answer; the violence section began with an acknowledgement that relationships sometimes have conflict, and a reminder of their option to skip items. GBV support services were embedded in a list of supports to minimise risk that they would cause alarm. Participants received KES500 or US$5 per survey completed.

Focus group discussions (FGDs) and individual in-depth interviews (IDIs) were conducted by trained interviewers among male and female youth participants and community stakeholders via the Zoom videoconferencing platform. FGDs were conducted in August 2020 with youth (eight FGDs; total n=64) and stakeholders working at youth-service community-based organisations (four FGDs; n=32). IDIs were conducted with cohort members following their survey completion at 12-month follow-up (n=20) and 18-month follow-up (n=20); purposive sampling sought representation across age, gender, pre-COVID activity (school or work), family planning and violence experience. All qualitative activities followed a semistructured interview guide that included, but was not limited to, risks for IPV and SV. The IDI design maximised breadth in perspective and point-in-time inputs; sampling at each wave was independent and did not prioritise longitudinal trajectories.

### Measures

#### Intimate partner violence

IPV was assessed with a shortened Revised Conflict Tactics Scale-2.^37^ Partnered participants were asked single items about physical partner violence (‘Has a partner pushed you, thrown something at you that could hurt you, punched or slapped you?’) and sexual partner violence (‘Have you had sex with a partner when you did not want to due to threats, pressure or force?’; assessed at mid-pandemic and late-pandemic only). At pre-pandemic, assessments referred to lifetime experiences with a current or former partner; 12-month follow-up (2020) and 18-month follow-up (2021) assessments ascertained past 12-month and past 6-month prevalence, respectively, both specific to a current partner. At 12-month follow-up (August–October 2020), participants reporting past-year IPV were asked about timing relative to COVID-19 restrictions, specifically: before COVID-19 restrictions only, since COVID-19 restrictions only, or both. Participants reporting IPV both pre- and since COVID-19 restrictions characterised changes in intensity since the pandemic restrictions (decreased, no change, increased).

IPV (physical and/or sexual) outcomes were constructed based on these assessments: (1) IPV experience at each time point, (2) IPV during the pandemic, indicated by presence of IPV since COVID-19 restrictions at 2020 and/or at 2021 survey and (3) IPV trajectory, a mutually exclusive categorical variable reflecting: sustained safety (no IPV at any time point), IPV cessation (IPV pre-COVID, resolving by 2021), IPV initiation (no IPV pre-pandemic, initiation at 2020 or 2021) and intermittent IPV (IPV pre-COVID and 2021 but not 2020 or 2020 but not 2019 or 2021).

Partnership was assessed with a single item; at pre-pandemic participants were asked if they were currently ‘involved with someone in a sexual or romantic relationship’. At subsequent rounds, participants were asked if they had a ‘sexual or dating partner’ in the last 12 months and 6 months, respectively (pre-pandemic 58.7%, 12-month follow-up 73.4%, 18-month follow-up 68.4%).

#### Non-partner SV

Non-partner SV was assessed at 12-month follow-up (past 12 months) and 18-month follow-up (past 6 months) via single item: ‘Have you had sex when you did not want to with anyone else (not a partner) due to threats, pressure, or force?’).

#### Violence-related help-seeking

Among AGYW who reported experiencing IPV or non-partner SV, a single item assessed help-seeking ‘Did you seek help for any experiences of harm or unwanted sex?’) at 12-month follow-up (2020) and 18-month follow-up (2021).

### Other study variables

Standard demographic assessments included age, education, subjective household socioeconomic status (SES),^38^ pre-COVID main activity (paid work vs school or caregiving), 2020 family structure (living with parents vs living alone, with partner or other people) and household prime earner (self vs another person). A three-item social support assessment was adapted from the Multidimensional Scale of Perceived Social Support (range 3–15) with lower scores indicating greater social support.^39^ Control over the decision to leave the house was assessed via 4-point Likert scale, with categories collapsed for analysis (0=‘none/very little,’ 1=‘some/a fair amount’ or 2=‘full control’).^40^

Dyad-level assessments for partnered AGYW included 2020 partner cohabitation status, age difference with current partner (dichotomised as ≤4 years difference and >4 years difference), transactional relationship (ie, started or continued a sexual or dating relationship in order to receive resources), and relationship fear (ie, tried to not cause problems with a partner over being afraid of what they might do).

Assessments of changes since COVID-19 restrictions at 2020 survey included changes in the amount of time at home and with their partners, respectively (dichotomised for analysis as less/unchanged or more), changes in personal control to leave the house (less, unchanged, or more), changes in economic reliance on others (more reliant vs not more reliant).^40^

Individual, dyad, and COVID-related assessments aligned with best practices; existing measures were used when possible, including from Evidence-based Measures of Empowerment for Research on Gender Equality.^40^ Likert scales were dichotomised based on underlying distributions to maximise statistical power.

#### Analytical samples

Non-partner SV analyses were conducted among all female participants at 12-month follow-up (2020) (n=612) and 18-month follow-up (2021) (n=591). IPV analyses were limited to partnered AGYW (n=550 prepandemic; n=449, 12-month follow-up; n=404, 18-month follow-up). Cross-sectional analyses were further restricted to those with complete IPV data (<3% missing) per time point. Analyses specific to IPV during the pandemic were limited to AGYW with 12-month follow-up (2020) or 18-month follow-up (2021) data (n=363) and IPV trajectory analysis was conducted with the subset who were currently partnered and who had complete IPV data at all three rounds (n=246). Attrition and partner status were non-differential to IPV reported at the last survey round.

### Analysis

#### Quantitative analysis

Prevalence of IPV, non-partner SV and related help-seeking, respectively, were calculated for each survey round for which data were available. At 12-month follow-up (2020), we characterise timing of violence, and changes in violence severity, relative to COVID-19 restrictions.

Prevalence of IPV at any point during the COVID-19 pandemic was calculated for the overall sample, and by individual, dyad and COVID-19-related factors assessed at 2020 survey; bivariate differences were evaluated by design-based F-statistics. Finally, a multivariable logistic regression model was constructed using generalised linear model (GLM) link log and family binomial. Factors associated with IPV bivariately at p<0.10 were included in the initial model. Due to collinearity among key variables, a theoretically informed manual stepwise model building process was used to fit a parsimonious model that accommodated key demographic variables.

IPV trajectories prior to and through the pandemic were first visualised using a Sankey diagram for AGYW who were partnered at all three time points (n=246). Categorical IPV trajectories (sustained safety, cessation, initiation, intermittent) were described and compared by individual, dyad, and COVID-related factors, with differences assessed via design-based F-statistics.

All analyses were conducted using Stata V.17.0. To mitigate potential biases introduced by the recruitment strategy, sampling weights accommodate the RDS study design using RDS-II (Volz-Heckathorn) weights, postestimation adjustment based on 2014 KHDS population data (age, sex, education levels) and lost to follow-up adjustment where relevant. Analysis adjusts for clustering by RDS seed chain at baseline.

#### Qualitative analysis

FGDs and IDIs were audiorecorded, transcribed verbatim and translated to English language (if needed). Resulting transcripts were coded using both inductive and deductive techniques; guided by gender theory^41^ and emergent literature on GBV during COVID-19. A subset of transcripts was first coded by two independent research assistants. Coding discrepancies were reviewed and reconciled; iterative changes were made to the codebook and applied to remaining transcripts as new themes emerged. GBV-related codes were extracted and organised into matrixes by sub-theme for analysis; sub-themes were synthesised across interview type and participant gender, and deviant cases were discussed. Finally, relevant quotations were compiled and reviewed by the research team. The present analysis sought to maximise the voices of young women.

## Patient and public involvement

This community-engaged study sought public and end-user input at all phases. During the formative research stage prior to the 2019 cohort recruitment, input from community-based, youth-serving organisations informed the study recruitment strategy for feasibility, survey measures and constructs to ensure relevance, and study logistics to maximise participant comfort and confidentiality. The qualitative data collection in 2020 generated refinements to survey content for 2021 round. All recruitment and procedures were conducted by trained REs selected from underlying communities, and who provided inputs on measures for clarity and aided in results interpretation. Findings were disseminated in November 2020 and again in September 2021 with stakeholders spanning policy sector, government representatives, elders/faith leaders, community-based organisations, and youth leaders from the study communities.

## Results

### Quantitative results

**Figure 1:**
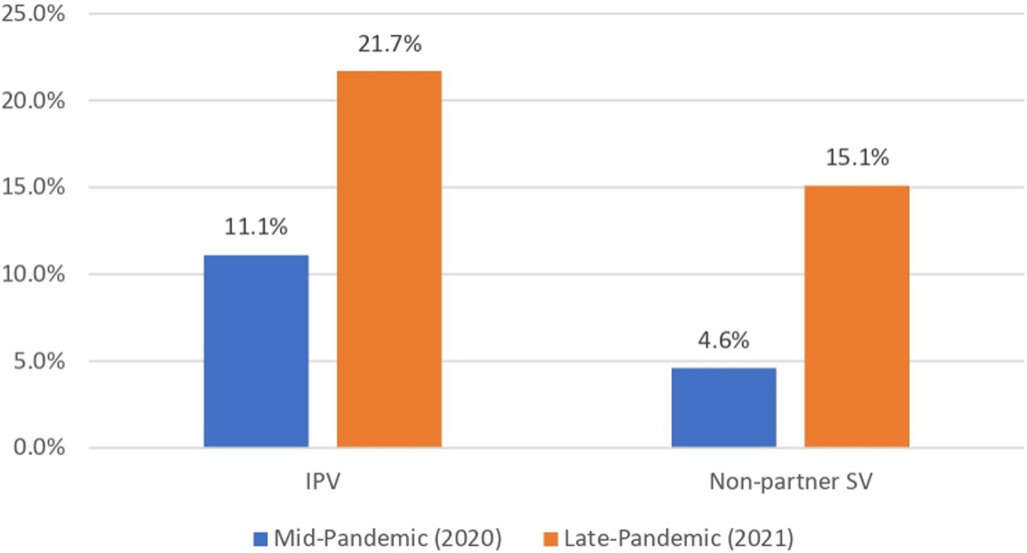
Percent* of women who experienced violence and sought help at each pandemic period, by violence type. *Weighted. IPV, intimate partner violence; SV, sexual violence.

IPV prevalence was 17.5% (lifetime) with current/recent partner at pre-pandemic (2019), 17.3% past-year at 12-month follow-up (2020), and 17.5% past-6 months at 18-month follow-up (2021) (table 1). Among AGYW reporting past-year IPV at 12-month follow-up, 43.3% experienced IPV only since the pandemic began. Non-partner SV prevalence was similar at 12-month follow-up (3.0%; past-year) and 18-month follow-up (3.1%; past 6 months). Among AGYW who experienced IPV, 11.1% sought help at 12-month follow-up (2020) and 21.7% sought help at 18-month follow-up (2021), respectively (figure 1). Similar increases in help-seeking across time points were seen among AGYW who reported non-partner SV (4.6% at 12-month follow-up; 15.1% at 18-month follow-up).

**Table 1:** Prevalence of past-year intimate partner violence and non-partner sexual violence at pre-pandemic (2019), 12-month follow-up (2020) and 18-month follow-up (2021), and changes since COVID-19 restrictions

	IPV	Non-partner sexual violence
	% (n*)	
Prepandemic baseline (2019)	n=550	
Prevalence with current/former partner†	17.5 (96)	−
12-month follow-up (2020)	n=449	n=612
Prevalence (past 12 months)	17.3 (78)	3.0 (18)
Timing relative to COVID-19 restrictions‡		−
Before-COVID-19 restrictions only	29.9 (20)	
Since COVID-19 restrictions only	43.3 (29)	
Both time periods	26.8 (18)	
Change in intensity since COVID-19 restrictions if experienced during both time periods§		−
Decreased	18.9 (4)	
No change	32.5 (6)	
Increased	48.6 (9)	
18-month follow-up (2021)	n=404	n=591
Prevalence (past 6 months)	17.5 (71)	3.1 (18)

−Item not measured at specified time point.

*Weighted.

†Item wording specifies ever violence experience with current or former (if no current) partner.

‡Among those with past 12-month IPV at 12-month follow-up (n=67 unweighted; n=78 weighted).

§Among AGYW who reported IPV both before and since COVID-19 pandemic (n=19 unweighted; n=18 weighted).

AGYW, adolescent girls and young women; IPV, intimate partner violence.

Among partnered AGYW, 27.6% experienced IPV during the pandemic (ie, at 2020 or 2021) (table 2). IPV during the pandemic was significantly more prevalent among AGYW with less than a secondary education (vs secondary education or higher; 42.6% vs 22.8%, p=0.006), in lowest relative household SES tertile (vs middle and highest tertiles; 40.0% vs 21.7% and 19.2%, p=0.001), whose main prepandemic activity was paid work relative to those in school or caregiving (33.9% vs 18.0%, p=0.01), who lived alone or with others (vs with parents; 38.7% vs 19.2%; p=0.001), who were primary household earners versus those supported (42.5% vs 24.6%, p=0.02), and those with low social support (46.0% vs 22.7%, p=0.003). At the dyad level, IPV during the pandemic was more common for those with partner age difference greater than 4 years (40.1% vs 22.0%, p=0.005), and less common among those in transactional relationships (20.1% vs 35.1%, p=0.02).

**Table 2:** Sample characteristics‡ overall and by intimate partner violence during COVID-19 pandemic (n=363*), weighted

	Overall	IPV during COVID-19 pandemic		
	%	Row %	P value†	aOR (95% CI)†§
Total	**−**	27.6		−
Individual				
Age group			0.48	−
16–20 years	30.4	31.1		
20–25 years	69.6	26.0		
Highest level of education completed			**0.006**	−
Secondary/‘A’ level or higher	76.1	22.8		
Less than secondary	23.9	42.6		
Subjective household SES tertile			**0.001**	−
Lowest	37.9	40.0		
Middle	20.5	21.7		
Highest	41.6	19.2		
Pre-COVID main activity			**0.01**	
School/caregiving	39.9	18.0		ref
Paid work	60.1	33.9		**1.72 (1.11 to 2.68**)
Family structure			**0.001**	−
Lives with parents	57.3	19.2		
Lives alone, with partner or others	42.7	38.7		
Primary earner			**0.02**	−
Self	16.4	42.5		
Someone else	83.6	24.6		
Social support			**0.003**	
High	79.1	22.7		ref
Low	20.9	46.0		**2.02 (1.47 to 2.76**)
Personal control to leave household			0.25	−
None or very little	22.3	20.7		
Some or a fair amount	39.3	25.7		
Full control	38.4	33.5		
Partner dyad				
Living with partner			0.42	−
No	82.2	26.3		
Yes	17.8	33.1		
Age difference with current partner			**0.005**	
<4 years difference	69.4	22.0		ref
>4 years difference	30.6	40.1		**1.95 (1.41 to 2.70**)
Past 12-month transactional relationship			**0.02**	−
No	36.8	35.1		
Yes	63.3	20.1		
Fear in relationship in 2019			0.12	−
No	53.9	24.6		
Yes	46.1	35.7		
COVID-19 impacts since onset of restrictions				
Ability to meet basic needs since COVID-19 restrictions			0.07	
Able to meet basic needs	44.0	21.2		ref
Not able to meet basic needs	56.0	32.6		1.30 (0.83 to 2.02)
Change in amount of time at home since COVID-19 restrictions				−
Home less/unchanged	12.5	36.0		
Home more	87.5	26.3		
Change in personal control to leave household since COVID-19 restrictions			0.66	−
Less control	29.2	28.0		
Unchanged	25.5	32.1		
More control	45.3	24.7		
Change in amount of time with partner since COVID-19 restrictions			0.64	−
Less time/unchanged	57.9	24.6		
More time	42.1	27.7		
Change in economic reliance on others since COVID-19 restrictions			0.14	−
More reliant	59.4	23.3		
Not more reliant	40.6	33.7		

−indicates not included in final adjusted model aOR.

*Among partnered AGYW with complete 12-month follow-up or 18-month follow-up data.

†P value from design-based F-statistic for IPV experience by sample characteristic boldface indicates statistical significance at p<0.05.

‡Characteristics obtained at 12-month follow-up (2020).

§Generalised linear model (GLM) with link log and family binomial, accounting for robust SE clustering by node and survey design weighting.

AGYW, adolescent girls and young women; aOR, adjusted OR; IPV, intimate partner violence; SES, socioeconomic status.

In adjusted analyses, IPV experience during COVID-19 remained associated with low social support (aOR 2.02; 95% CI 1.47 to 2.76); partner age difference >4 years (aOR 1.95; 95% CI 1.41 to 2.70) and pre-COVID main activity of paid work (vs school or caregiving; aOR 1.72 (95% CI 1.11 to 2.68).

**Figure 2:**
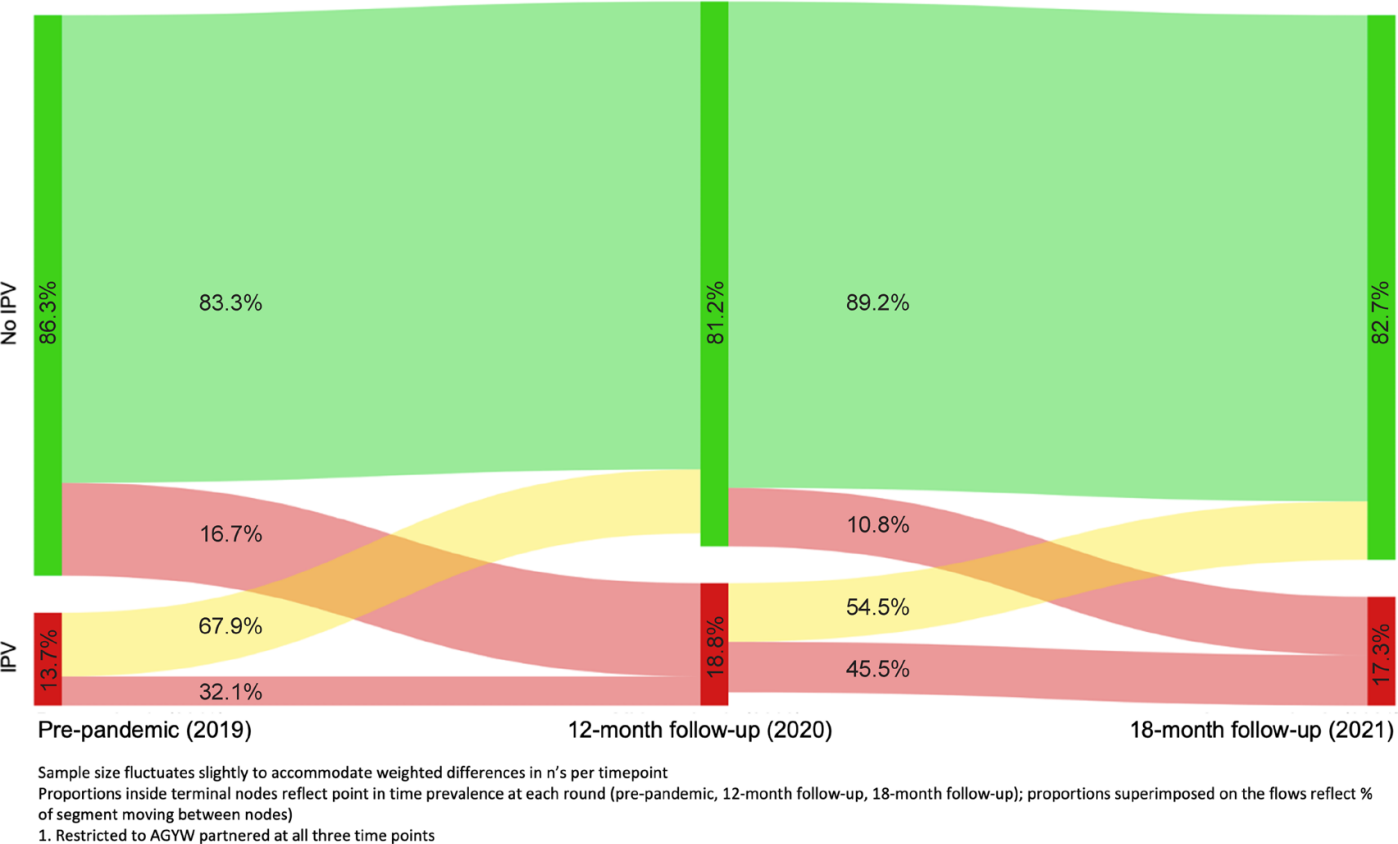
Trajectories of intimate partner violence from prepandemic (2019) to mid-pandemic (2020) to late-pandemic (2021) (n=246 AGYW, weighted). AGYW, adolescent girls and young women; IPV, intimate partner violence.

Figure 2 illustrates transitions in IPV across pre-pandemic (2019), 2021 and 2021 surveys among AGYW who were partnered at all three time points (n=246). In this subset, approximately one-third (32.1%) of those with pre-pandemic IPV also indicated past-year IPV at 12-month follow-up (2020). In 2020, past-year IPV prevalence was 18.8%, including 16.7% of AGYW who indicated new IPV since the pre-pandemic survey wave. Between 12-month (2020) and 18-month follow-up (2021), the majority of AGYW who indicated no IPV at 2020 remained in that group (89.2%), while 10.8% indicated new IPV by 2021. Among those with past-year IPV at 2020, 54.5% transitioned to no IPV and 45.5% remained exposed to IPV in 2021. At each time point, a meaningful proportion transitioned from IPV to safety (67.9% of those with IPV at pre-pandemic indicated no IPV by 2020; 54.5% of those with IPV at 2020 indicated no IPV by 2021).

Among AGYW who were partnered at all three time points (n=246), 66.2% had sustained safety (no IPV) across all three time points, 9.2% transitioned from pre-COVID IPV to safety (cessation), 11.1% experienced new IPV during the pandemic (initiated) and 13.6% indicated intermittent IPV (table 3). Trajectories varied by education level (p<0.001), pre-COVID main activity (p=0.04), social support (p=0.02) and age difference with partner (p=0.02). Intermittent IPV was as high as 32.7% of AGYW with less than secondary education, 18.1% of AGYW doing paid work pre-COVID, 30.2% for those with low support and 24.7% for those with >4 year age difference with partner. Among AGYW in transactional relationships, intermittent IPV was somewhat lower than average (7.1%) and IPV cessation somewhat higher (12.0%); p=0.02.

**Table 3:** Intimate partner violence trajectories over 18 months prior to and during the COVID-19 pandemic, by 2020 survey characteristics (n=246†, weighted)

	Sustained safety (n=162)	Cesate IPV (n=23)	Initiate IPV (n=27)	Intermittent IPV (n=33)	P value*
	%	%	%	%	
Overall	66.2	9.2	11.1	13.6	
Individual					
Age group					0.61
16–20 years	62.2	7.8	10.3	19.7	
21–26 years	67.7	9.7	11.3	11.3	
Highest level of education completed					**<0.001**
Secondary/ ‘A’ level or higher	72.8	8.3	10.7	8.2	
Less than secondary	42.6	12.5	12.3	32.7	
Relative household SES tertial					0.27
Lowest	52.5	9.3	15.7	22.5	
Middle	69.3	9.0	8.3	13.4	
Highest	73.9	9.2	9.3	7.6	
Pre-COVID main activity					**0.04**
School/caregiving	78.3	6.6	8.1	7.0	
Paid work	57.8	11.0	13.1	18.1	
Family structure					0.10
Lives with parents	73.1	9.1	7.1	10.7	
Lives alone, with partner or others	56.0	9.3	17.0	17.8	
Primary earner					0.37
Self	53.3	11.4	13.4	21.9	
Someone else	69.1	8.8	10.5	11.7	
Social support					**0.02**
High	71.3	9.5	9.7	9.5	
Low	45.0	8.0	16.8	30.2	
Personal control to leave household					0.37
None or very little	65.1	16.5	7.6	10.8	
Some or a fair amount	69.9	3.4	10.1	16.7	
Full control	62.2	11.5	14.9	11.4	
Partner dyad					
Living with partner					0.32
No	67.3	9.2	8.8	14.6	
Yes	61.6	9.0	20.3	9.2	
Age difference with current partner					**0.02**
<4 years difference	73.3	8.6	9.4	8.7	
>4 years difference	49.9	10.5	14.9	24.7	
Past 12-month transactional relationship					**0.02**
No	60.2	3.9	10.0	25.9	
Yes	69.4	12.0	11.6	7.1	
Fear in relationship in 2019					0.06
No	75.7	4.6	6.9	12.8	
Yes	55.8	14.1	15.4	14.7	
Fear in relationship in 2020					0.44
No	70.9	8.6	11.4	9.1	
Yes	60.4	9.2	11.5	18.9	
Changes relative to start of COVID-19 restrictions					
Change in ability to meet basic needs					0.07
Able to meet basic needs	72.6	10.1	12.1	5.2	
Not able to meet basic needs	61.4	8.5	10.2	19.8	
Change in amount of time at home					0.13
Home less/unchanged	61.0	7.2	26.9	4.9	
Home more	66.9	9.5	8.9	14.8	
Change in personal control to leave house					0.64
Less control	67.5	7.9	10.2	14.6	
Unchanged	64.7	8.6	5.8	21.0	
More control	66.2	10.4	14.4	9.0	
Change in amount of time with partner since COVID-19					0.17
Less time/unchanged	70.9	6.8	12.5	9.2	
More time	58.6	12.8	9.5	19.1	
Change in economic reliance on others					0.22
More reliant	71.2	7.0	11.7	10.1	
Not more reliant	57.9	12.9	9.9	19.4	

*P value from design-based F-statistic; boldface indicates statistical significance at p<0.05.

†Restricted to AGYW who were partnered at all three rounds.

AGYW, adolescent girls and young women; BD, put all three references here for the data; IPV, intimate partner violence; SES, socioeconomic status.

### Qualitative results

Qualitative themes included impact of the COVID-19 mandated restrictions, pandemic-related psychological and financial stress, and endemic conditions that give rise to violence.

The COVID-19 restrictions on IPV risks were often shaped by marital/cohabitation status. Among those residing at home, being out past curfew enabled vulnerability to IPV.

According to me, conflict in relationships like now there are curfew hours so maybe you went out and time really went, time went by without you noticing and you remained there and your parents didn’t know where you went. And now we come back to the relationship, you are with this guy and now he has the chance to do with you anything he likes because he knows you can’t go anywhere. It is past curfew, you can’t leave the house. So, he might do anything to you (Young woman, age 15–19 years).^42^

For other AGYW, particularly those still living with parents, restrictions allowed them to avoid or end violent relationships.

According to me, it has affected positively. Because before, the violence was there so much but nowadays it is hard to meet, and when you meet it is hard. So, if there is any violence which comes up, you break up (Young woman, age 15–19 years).^42^

Among cohabitating couples, increased time at home with a partner due to COVID-19 restrictions increased potential for conflict and in turn, IPV.

Eehe (meaning yes) [violence] has increased let’s say in this family the guy is the type who likes to get annoyed so fast, you know before he could leave to work so there would be no much quarrels. You know right now they are in the house together all through so right now it is fights every time (Young woman, age 15–19 years).

Further, inability to leave the home due to curfew hindered ability to escape to safety when experiencing violence.

So, you find any quarrel, the female becomes subjected to violence but can’t go out because it’s curfew time, she can’t go anywhere so she is patient with the beatings, so you find violence is there, it affects more the female than the male partner (Young man, age 20–24 years).

Pandemic-related restrictions reduced IPV risk in indirect ways; specifically, curfews were described as reducing partners’ alcohol consumption, thus lowering the risk of violence.

For relationship[violence] it has reduced, why? Like let’s say like the way men used to like to stay out in bars, they stay there for long. So, right now where will they go? Coz (because) let’s say curfew is nine, they will be at home at nine…Whereby there will be no conflicts, conflict there up and down (Young woman, age 15–19 years).

Okay people’s husbands because we have at this age, our age 15–24, we have people who have already have married. So, their husbands are getting home earlier and if they are getting home earlier, there are things like beer, people are not drinking since that, drinking time is at night if you tell a person that you are drinking at one during the day that is not lunch. So, it is happening like many people are now sober. And you know when people are sober, violence reduces (Young man, age 20–24 years).

Psychological and financial stress related to pandemic-induced job loss was further described as an indirect risk for increased IPV in the home.

We can see now gender-based violence coming up especially for the young couples […] There is a lot of emotional and psychological stress that has come with Covid so their relationships have been affected so much and with loss of employment. Their financial status is not very stable. So that also has affected their relationship (Stakeholder).

So in the family you find that let’s say the boy or father was the bread winner in the house. So due to lack of money, we know not all have money, poverty brings a lot of things like conflicts, so you find there are disagreement in the house so you find there will be fights (Young woman, age 15–19 years).

The economic strife of COVID-19 also increased IPV risk by threatening traditional gender norms that position men as primary earners within the household/family.

Conflicts have also increased in a way because there are no finances in the house. You know men really like being on the upper limit because I am giving out the money you know and now he doesn’t have money so the slightest thing in the house makes him angry so this has really led to conflict in the house (Young woman, 15–19 years).

Like, like right now, like men are the ones who mainly provide and since corona came, many people lost their jobs a lot. So, such a man was the one providing. Like job is over, right now he is not providing, so, it brings conflict at home (Young man, 20–24 years).

Endemic risk factors for relationship violence were also described, including alcohol and financial conflict unrelated to the pandemic.

[relationship violence] is fueled by drunkenness (alcohol). At the household, financial issues, lack of money contributes to it. Because maybe you want food but the partner does not have money. So he ends up getting upset, you do not understand him, you start quarreling (Young woman, 20–24 years).

I am the one who started because he was not supporting the child. He thought I have another man. So, I started avoiding him. I told him that it is not a must for him to support the child. He got angry. When he got angry, he thought that I have my own things and that I want to play him. He called me and I refused to go. He called me for two weeks and I refused to go. The third week he came to quarrel here at our home…So, he came and quarreled and I went out. Now, I do not know whether he had drunk or what was wrong. So, it look like a fight. He told me to give him his child and I refused. So, it was like a fight… It was a fight because he hit me (Young woman, 15–19 years).

Notably, young women did not discuss personal experiences seeking help for violence. In reflecting on GBV services, stakeholders and key informants described a cascade of barriers including gaps in knowledge, COVID-19 related closures and stigma.

Yeah, so I think the…the places are there, there are facilities that offer such kind of ah…assistance. However, there is still room to improve in terms of information dissemination and knowledge of ah…the youth in terms of where they can access the services. (Stakeholder)

I think with this CovidCOVID-19 and you know some of the safe houses have been closed it is difficult for them to access the services or the safety spaces that were opened before the pandemic. (Key Informant)

Social stigma related to violence reporting also limited AGYWs’ ability to seek help.

Remember also not everyone who will be assaulted will go to the facility. There is also some element of stigma with it. So, whether there is COVID or not those who would have gone to the facility to seek health services after having experienced domestic violence, they will still go. And those who will not go because of issues of stigma still they won’t. When it comes to the services, the services have been there and they are still there (Key Informant).

Additional perceived barriers to help-seeking included the climate of impunity for violence against women, as well as fear of retribution.

Yes, I think young people are aware where to report [GBV] but I think we need to strengthen something eh. Because when these persons report and nothing happens, then this persons gets fear because if he was reporting that he was being… there is an incest, there is a rape and nothing happens and then the person who had done that is still loitering within the community, the society, then there is that fear of insecurity to this persons who has reported. (Stakeholder)

## Discussion

Findings from this first prospective study of GBV trajectories among AGYW in Nairobi, Kenya demonstrate striking consistency in IPV prevalence (~17%) prior to and during the COVID-19 pandemic. Over one in four (27.6%) AGYW were affected by IPV at some point during the pandemic by the 2021 data collection round. By contrast, non-partner SV was far less prevalent, limiting trajectory analysis. Current results from this sample of primarily non-cohabitating urban AGYW expand a global dialogue primarily focused on IPV-related pandemic impacts among cohabitating couples, and affirm risk of violence to AGYW during public health emergencies. The relative consistency in IPV prevalence from pre-COVID through the pandemic argues for recognition of endemic violence against AGYW. IPV prevention and response investments must be prioritised during COVID-19, and sustained beyond this global health emergency. Recovery efforts must address pandemic-related needs for safety, while working to overcome the normative, social and economic systems that give rise to IPV and other forms of violence against AGYW.

Against the dearth of prospective data on IPV trajectories for AGYW in Nairobi and elsewhere, the IPV-related transitions in and out of safety among AGYW across the three time points (figure 2) are highly informative. AGYW’s trajectories spanned IPV cessation, initiation and intermittent experiences, illustrating fluidity both in and out of relationship safety. These patterns likely reflect variability in triggers for violence, though in the absence of comparable data from non-emergency periods, it is difficult to discern how much variability is attributable to pandemic impacts as compared with natural trajectories in AGYW relationships. Qualitatively, participants discussed endemic conditions that contribute to violence as well as COVID-19-related factors that can exacerbate relational conflict and lead to violence. Though increases in reported household conflict since the start of the pandemic have been observed cross-sectionally in other settings in Kenya and globally,^21^ among our sample, the COVID-19-related factors assessed were not associated with IPV during the pandemic nor with IPV trajectories. These data urge consideration of the underlying relational and power dynamic factors that give rise to IPV for AGYW in this setting. Because it is possible that the COVID-19 changes and their impact on IPV fluctuated in a way that was not captured due to the timing or sensitivity of assessments, further research is needed to better understand the ways in which pandemic experiences impacted AGYW safety.

The socioeconomic patterning of IPV for AGYW was complex. Economically vulnerable AGYW, that is, those with low household SES and having less than secondary education, had higher IPV risk. A cross-sectional study among women in three counties in Kenya found a similar relationship between domestic violence and SES.^43^ Simultaneously, elevated risk of IPV during COVID-19 was identified among AGYW who were primary earners and/or engaged in work prior to COVID-19. While these economic indicators may suggest protection through economic leverage, their income and earnings may not have been sufficiently protective against IPV due to persistent gender gaps in earnings, earning potential and economic opportunities. Their economic endeavours may have threatened underlying gendered systems that traditionally position men as breadwinners, as suggested by qualitative evidence. Early evidence suggests economic empowerment programmes can reduce violence against young women even during the pandemic^44^; this strategy may represent a meaningful solution to the identified IPV risk to economically vulnerable AGYW, particularly given underlying gender earning gaps in Nairobi and elsewhere, coupled with the detrimental cascade impact to women’s social and economic opportunity incurred by COVID-19.

The increases in help-seeking for both IPV and non-partner SV observed from 12-month follow-up (2020) to 18-month follow-up (2021) are promising, particularly as violence-related help-seeking is low in Kenya and globally, particularly for formal supports.^19^ Stakeholders and key informants noted multiple barriers to help-seeking, including knowledge gaps and stigma. COVID-19 related closures were also noted as a potential gap in accessing care. Social norms and gendered social systems can create a culture of IPV tolerance and stigma that challenges women’s ability to seek help or identify their experiences as abuse. These same forces can stigmatise and shame young people engaged in premarital relationships, particularly sexual relationships, further inhibiting their ability to get help. The relative increase in help-seeking from 12-month follow-up (2020) to 18-month follow-up (2021) seen in our quantitative results may reflect increased public awareness and normalisation of support services during the COVID-19 pandemic and in the wake of a government-launched investigation in 2020.^22^ Further, young women may have begun to access formal services and informal support of friends and family as mandated restrictions and curfews were lifted in Nairobi. As awareness and use of local supports continues to grow, there remains a great need to communicate accessibility to AGYW and overcome potential barriers to care, including stigma, and modes of dissemination during times of limited travel and social gathering should be considered. Maintaining GBV-related services during emergencies, communicating their availability and normalising their use are needed to lift stigma and ensure access to care. Strikingly, social support was protective against IPV during the pandemic, and those with low social supports were at highest risk for intermittent IPV in trajectory analysis. While the source of social supports are not known, these results do affirm the value of connectivity and support in buffering against IPV risk for AGYW.

The relatively low prevalence of non-partner SV (~3%) may be considered surprising, given local reports of increased violence during the pandemic. However, national data from Kenya illustrate that intimate partners are the primary perpetrators of SV against young women,^20^ as in other settings, thus it is possible that the current non-partner SV estimates are accurate for AGYW. Under-reporting due to stigma or social desirability is a possibility; it is also possible that measures were not sufficiently sensitive.

The current levels of violence against AGYW are alarming yet highly actionable, even in pandemic conditions. The COVID-19 pandemic galvanised recognition of GBV globally and in Kenya, creating the necessary policy window for meaningful prevention and response. In May, 2021, Kenya president Uhuru Kenyatta pledged an unprecedented investment of US$23 million by 2022 and a further US$50 million by 2026 address GBV.^45^ WHO guidelines for violence assessment and response can be integrated within community programmes and clinics serving AGYW^46^; during pandemics and related emergencies, these strategies can be embedded in testing or support facilities. Technology-based solutions offer the added advantage of accessibility even during mobility restrictions throughout the COVID-19 and future public health emergencies; technology-based relationship safety assessment and planning tools have been found acceptable and valuable for safety planning and connection to care within Kenya,^47^ and can be implemented more widely to reach AGYW even in emergencies. Further, the disruption of the COVID-19 pandemic represents a window of opportunity to disrupt the harmful social norms that maintain tolerance of IPV and other forms of violence, and the gendered economic imbalances that render women dependent on potentially harmful partners. In other disrupted settings, strategic community mobilisation interventions have generated potent social norms change including reductions in justification of abuse and increased confidence in service provision^48^; similar interventions may be valuable if mobilised by urban youth. Postpandemic rebuilding must harness the GBV-related awareness created by the pandemic, to prioritise gender equity, destigmatise violence, scale evidence-based prevention and normalise access to services and meaningful support for violence survivors, particularly youth.

Results should be understood in light of several limitations. Two key measurement issues limit precision. First, the referent time periods for the IPV assessments vary, and only two of the three represent true, time-bound period prevalences, limiting comparability. Second, the IPV and SV measures were single assessments to limit survey length and minimise risk to participants; single items and short-form assessment are less sensitive,^49^ introducing risk for misclassification, particularly underreporting. Under-reporting of IPV and non-partner SV, particularly for interviewer-administered waves, also could stem from social desirability biases and privacy concerns, despite extensive training and privacy protocols aligned with best practices. Generalisability to the underlying youth population in urban Nairobi is enhanced through RDS and postestimation weights; while the cohort was recruited without mobile phone ownership eligibility criterion, it is possible that the phone-based interviewing during the pandemic waves of data collection contributed to a higher SES sample, even despite the high mobile connectivity of Nairobi. Small cell sizes limit precision of estimates as well as advanced analytic techniques, particularly regarding non-partner SV and understanding changes in intensity of IPV through the pandemic period. Measures did not characterise severity nor chronicity of experiences, as is needed to better inform prevention and response. Despite the value of the qualitative learnings, the tools were not focused solely on GBV, which limits their depth for this topic. Pandemic restrictions limited time with quantitative and qualitative participants, and total qualitative sample size, limiting depth and nuance on this topic.

The COVID-19 pandemic has far-reaching implications for global health and safety. AGYW in Nairobi, Kenya remain at risk for IPV during COVID-19, reflecting a combination of pandemic-specific influences together with underlying social and economic disparities rooted in gender inequality. Simultaneously, AGYW stand to gain the most from effective prevention and response, as disrupting violence early may interrupt longer-term cycles of harm. Sustained, comprehensive investments in GBV prevention and response are needed to ensure progress toward the Sustainable Development Goal of elimination of violence against women, and its positive cascade impact on social, economic and well-being of women and families for generations to come. Effective prevention and response programming must be sustained during emergencies and scaled, with monitoring and impact evaluation to ensure goals are met. Essential steps include ending stigma and silence on GBV for AGYW and facilitating a culture of help-seeking and survivor-centre support, particularly for youth.

## Data Availability

Data are available upon request from pmadata.org.
